# Biomarkers to Predict the Success of Treatment with the Intravitreal 0.19 mg Fluocinolone Acetonide Implant in Uveitic Macular Edema

**DOI:** 10.3390/pharmaceutics14040688

**Published:** 2022-03-22

**Authors:** Lucy Joanne Kessler, Grzegorz Łabuz, Gerd U. Auffarth, Ramin Khoramnia

**Affiliations:** 1 Department of Ophthalmology, University Hospital Heidelberg, 69120 Heidelberg, Germany; lucyjoanne.kessler@med.ui-heidelberg.de (L.J.K.); grzegorz.labuz@med.uni-heidelberg.de (G.Ł.);gerd.auffarth@med.uni-heidelberg.de (G.U.A.); 2 HEIKA–Heidelberg Karlsruhe Strategic Partnership, Department of Ophthalmology, Heidelberg University, 69120 Heidelberg, Germany

**Keywords:** uveitis, fluocinolone acetonide, macular edema, OCT biomarkers, choroidal vascularity index

## Abstract

To predict the need for additional local corticosteroids after receiving the 0.19 mg fluocinolone acetonide (FAc) implant in patients with macular edema secondary to non-infectious uveitis previously treated with local peribulbar corticosteroids. The number of corticosteroids required prior FAc, visual acuity, central retinal thickness, ellipsoid zone reflectivity ratio (EZR), and choroidal vascularity index (CVI) were compared between patients who did and did not require additional corticosteroids after FAc implantation. Pearson’s correlation coefficient (R) between putative predictors and the number of adjunctive corticosteroids after FAc implantation were measured; significant candidates were included in a generalized regression model. Patients who required additional corticosteroids after FAc had higher CVI and central retinal thickness as well as worse EZR at subsequent visits (*p* < 0.05). The number of corticosteroids required prior to FAc implantation (R: 0.49), CVI change from baseline to 6 months (R: −0.41), and central retinal thickness at baseline (R: −0.36) correlated to the number of additional corticosteroids (all *p* < 0.05). A higher number of corticosteroids per year before FAc implantation was predictive for an increase in corticosteroids required after FAc (odds ratio = 2.65), while a decrease in CVI from baseline to 6 months was inversely correlated (odds ratio = 0.82). Our results suggest that the more corticosteroids prior to FAc and the greater the short-term CVI reducing effect, the less is the chance to get additional corticosteroids after FAc.

## 1. Introduction

Uveitis is a chronic sight-threatening disease that can potentially lead to vision loss. The prevalence varies worldwide between 58 and 121 per 100,000, depending on geographic location and ethnicity [1]. Women have a higher prevalence among all age groups, likely due to the higher prevalence of autoimmune and rheumatic diseases in women who can develop non-infectious uveitis as an ocular manifestation of the underlying systemic disease [2,3]. Treatment options for non-infectious uveitis range from systemic therapy with immunosuppressive or disease-modifying anti-rheumatic drugs such as corticosteroids, methotrexate, or cyclosporine to local corticosteroid therapy for ocular manifestation or a combination of both [4]. Treatment intensity depends on the systemic and ocular disease activity, individual risk profile, and occurred adverse events. Corticosteroids (CS) are an important part of the treatment of uveitis as CS can provide rapid control of acute inflammation. CS can be given systemically or locally as topical eye drops, periocular, or intravitreal injections. Applying CS as an intravitreal sustained drug-delivery system can alleviate treatment burden and enhance patient compliance with lower injection frequency. In several European countries, both the 700 µg biodegradable dexamethasone (DEX) implant (OZURDEX; Allergan, Inc., Irvine, CA, USA) and the 0.19 mg fluocinolone acetonide (FAc, ILUVIEN; Alimera Sciences Inc., Alpharetta, GA, USA) are approved for the treatment of non-infectious uveitis affecting the posterior segment of the eye. The 0.19 mg FAc implant consists of a non-biodegradable polymer with a length of 3.5 mm and a diameter of 0.37 mm. FAc is a synthetic fluorinated glucocorticoid receptor agonist with no mineralcorticoid activity [5]. The Fluocinolone Acetonide for Diabetic Macular Edema (FAME) A and B randomized clinical studies showed the FAc implant was effective in patients with diabetic macular edema (DME) for up to 36 months; thus, the FAc implant was licensed for this indication [6]. Due to potent long-term inflammatory control in diabetic macular edema, the FAc implant was first used as an off-label treatment in patients with non-infectious uveitis. The efficacy of FAc in non-infectious macular edema was demonstrated in several two- and three-year clinical trial results [7,8]. These results led to the approval of ILUVIEN^®^ in several European countries so that the injectable 0.19 mg FAc implant became an alternative to dexamethasone implants for relapse prevention in non-infectious uveitis affecting the posterior segment of the eye. Notably, DEX and FAc have different pharmacokinetics. The DEX is a short-acting agent with high-rate drug release in the first 2 months followed by a low-dose release for a further 4 months. The FAc implant releases a low (0.2 µg/day) dose of fluocinolone acetonide in a nearly zero-order manner over a 36-month period [9,10,11,12]. Previous studies showed that the continuous release of low-dose CS leads to fewer fluctuations in central retinal thickness (CRT) in patients with diabetic macular edema and suggested that low-dose CS, as in the case of the FAc implant, might be more suitable for the long-term management of chronic inflammation in the eyes of patients with diabetic macular edema [13,14]. Several randomized controlled trials and real-world studies confirmed reduced recurrence rates increased recurrence-free periods and fewer adjunctive treatments compared to patients not receiving the FAc implant [8,15,16], thus confirming the long-term inflammatory control following FAc injection. A review of the literature confirmed that there are a small number of reports to have assessed the long-term clinical and morphological effects of the FAc implant or to have compared outcomes achieved with DEX and FAc implants [8,15,16,17,18,19].

Because individual treatment responses and the need for adjunctive CS treatment can be complex to understand due to heterogeneity of the underlying uveitis disease and individual inflammatory profile, recurrence-free periods remain hard to predict. Given the noninvasive and time-efficient nature of optical coherence tomography (OCT), it has become widely used for evaluating responses to therapy. So far, anatomical outcomes were mostly described by central retinal thickness or volume quantified in OCT. Emerging evidence for the role of other OCT biomarkers such as the ellipsoid zone reflectivity ratio (EZR) and choroidal vascularity index (CVI) for assessment and prediction of treatment response indicates a new promising approach to improve disease management in uveitis. The ellipsoid zone is thought to be formed by mitochondria in the inner segment of photoreceptors, thus representing the vitality of these cells [20]. Several authors demonstrated the correlation between EZR and visual acuity (VA) in retinal diseases associated with macular edema resulting from diabetic retinopathy or retinal vein occlusion [21,22,23]. EZR alteration during active uveitic inflammation and recovery after resolution has been described for several uveitis entities such as multiple evanescent white dot syndrome, punctate inner choroidopathy, and acute zonal occult outer retinopathy [24,25,26]. However, the longitudinal assessment of EZR in FAc-treated uveitis has not been conducted so far. CVI has been proposed by Agrawal et al. as a novel OCT biomarker to monitor uveitis activity, and since its introduction, CVI has become a major OCT biomarker in uveitis; however, it has not yet been evaluated in uveitic macular edema (UME) treated with the FAc implant [27]. Hence, the objective of the present study was two-fold: (1) to investigate the clinical and morphological changes, including the OCT biomarkers EZR and CVI, before and after FAc implantation; and (2) to compare outcomes in patients that did and did not require additional CS treatment following treatment with the FAc implant. The aim of this study was to find out potential predictive OCT biomarkers for additional local corticosteroid therapy after FAc implantation as whether treatment burden (frequency of local corticosteroid injection) can be reduced after FAc is one of the driving questions patients have and one of the proposed advantages of the FAc implant compared to short-acting alternatives.

## 2. Materials and Methods

### 2.1. Study Participants

This is a retrospective study including 29 participants who had a history of at least 1 year of non-infectious uveitis with macular edema requiring local treatment (topical, periocular, or intravitreal) as defined by the Standardization of Uveitis Nomenclature Working Group. At least one DEX implant or two periocular CS injections within 24 months before FAc implantation were used as inclusion criteria, and this allowed the comparison of structural changes under FAc versus other local injectable CS treatments. In cases of bilateral implantation of FAc, the eye with the longer follow-up time was chosen in this study. Retreatment with CS after FAc implantation was administered in case of recurrent UME (worsening of macular edema or the onset of macular edema). Patients with FAc implantation with a follow-up time of less than 6 months were excluded. Other exclusion criteria were the (absolute) spherical equivalent of more than 6 diopters, patients aged <18 years, and concomitant retinal diseases associated with macular edema. The effectiveness and safety results from 26 of the 29 patients reported here have been published previously [28].

### 2.2. Data

The electronic charts of all the patients who underwent FAc implant for non-infectious uveitis between January 2015 and February 2021 were reviewed. Clinical and OCT data were reviewed at 24, 18, 12, 6 months before and after FAc implantation and at treatment initiation (0 months/baseline), resulting in a maximum of nine time points spanning over 4 years. From 25 patients, complete clinical and OCT data were available for the two years before FAc implantation. From 23 patients, clinical and OCT data were available for at least 18 months after FAc implantation. Fourteen patients completed 2 years of follow-up. At each visit, patients completed measurement of best-corrected VA, tonometry, slit-lamp biomicroscopy, and indirect funduscopy performed by an experienced ophthalmologist specializing in uveitic diseases.

### 2.3. Image Acquisition

Images were obtained using the Spectralis Spectral Domain OCT with HeyEx software, version 5.3.3.0 to 6.9.4.0 (Heidelberg Engineering GmbH, Heidelberg, Germany). A 20° × 20° macular cube line scan protocol was used to obtain the image data. The first recorded scan was set as reference to ensure colocalization of all subsequent OCT scans. The horizontal B-scan through the foveola was extracted for further analysis. CRT was obtained from the built-in analysis tool of the proprietary software. OCT images with signal-to-noise ratio below 10 (with 35 being the best signal-to-noise ratio) were excluded from the analysis.

### 2.4. Image Processing and Analysis

For image preprocessing and analysis, we followed the protocols for EZR and CVI that have been published previously [21,29]. Logarithmic-transformed display of OCT was exported as tagged image file format (TIFF) using the integrated proprietary software. Semi-automated quantification of all three OCT biomarkers in the open-source software Fiji (U.S. National Institutes of Health, Bethesda, USA, https://imagej.net/software/fiji/ (last accessed on 27 January 2022)) has been described in the literature [27,30,31,32]. All images underwent a preprocessing of image registration for alignment and histogram-matched normalization before quantification. Regions of interest (ROI) was defined as the central 3 mm area. Briefly, for EZR, a longitudinal reflectance profile was obtained at every 200 µm. Ellipsoid zone reflectivity ratio (EZR) was calculated as the ratio of EZ reflectivity to RPE reflectivity, which is considered the most hyperreflective band in OCT. For quantification of CVI, the grader was masked to the time point of the OCT acquisition for manual selection of the region of interest. Hence, the grader did not know whether the OCT was taken before or after FAc implantation.

### 2.5. Statistical Analysis

First, characteristics and changes of outcome variables (visual acuity and OCT biomarkers) of the entire cohort and subgroups (patients with or without additional local CS after FAc) were described. Descriptive statistics of the entire cohort were reported as frequency and percentage for categorical variables and mean ± standard deviation for continuous variables. To describe the changes of outcome variables of the entire cohort during the observation time, paired-sample *t*-test or Wilcoxon signed-rank test, depending on the data distribution, were applied. Data from month 24 were excluded from pairwise comparison due to much smaller sample size (*n* = 14), which might reduce the statistical power. For subgroup comparison, the Mann–Whitney U test was applied. Adjusted *p*-values resulted from Bonferroni–Holm correction for multiple testing. Next, the relationship between adjunctive CS before and after FAc, and OCT biomarkers was evaluated. Pearson’s *Χ*^2^-test was used to test for correlation between CS injections before FAc implantation (≤2 or >2 CS/year) and the need for adjunctive CS after FAc. The effect size of this test was determined with Cramer’s V. The linear relationship between the number of CS injections after FAc, and structural and functional biomarkers was evaluated using the Pearson correlation coefficient. After a significant relationship between OCT biomarkers and the need for CS was found, we applied a generalized Poisson regression model, which is suitable for counts-based data, to study putative baseline predictors at the beginning of FAc therapy for the number of required adjunctive CS. As a regression model should not be inflated with a large amount of covariates, combinations of different covariates (OCT biomarkers at baseline and their changes from baseline to 6 months) were compared using the Akaike information criterion to define the best fitting model. Statistical analysis was performed in IBM SPSS Statistics software version 27.0 (IBM Corp., Armonk, NY, USA); a two-sided *p* < 0.05 was taken to represent a statistically significant difference. Adjusted *p*-values are reported where multiple testing was conducted. Decimal VA was converted to logarithm of minimal angle of resolution (logMAR) for statistical calculation.

## 3. Results

### 3.1. Clinical Characteristics and Comparison of Injection Frequencies

Study patients had a mean age of 60.03 ± 16.01 at the time of FAc injection, 34.50% (n = 10) were male, and the right eye was studied in 38.0% (*n* = 11) of cases. Patients were switched to FAc after a mean of 8.55 ± 6.20 years of disease duration. The mean observation time was 3.51 ± 0.51 years. Patients’ characteristics are described in Table 1.

Sixteen of 29 patients (55%) did not require any additional local CS for up to two years after FAc implantation. Among these patients, 81% (*n* = 13) had no more than two local CS injections per year before FAc implantation. In contrast, among those 13 patients who required additional local treatment, 10 (77%) needed more than two CS treatments per year before FAc implantation (effect size: 0.582 with a degree of freedom = 1, adjusted *p* = 0.006) [33]. An odds ratio of 0.069 (95% CI: 0.011 ± 0.419) indicated that ≤2 local corticosteroid injections before FAc implantation was inversely associated with additional injections after FAc; a patient who requires more than two injections/year before FAc has a 93.10% reduction in the chance of staying local CS-free after FAc implantation.

### 3.2. Observed Adverse Events after FAc Implantation

Long-term exposure to corticosteroids can lead to ocular complications, including cataract formation or elevated intraocular pressure (IOP), which can result in optic nerve atrophy. A total of 24 of 29 patients did not have any IOP-lowering medication prior to FAc implants. Among them, six patients developed elevated IOP between 21 and 30 mmHg and therefore received IOP-lowering eye drops. In all six patients, IOP returned to the normal range below 21 mmHg under local medication. No IOP-lowering surgery was required. Five patients were on IOP-lowering eye drops prior to FAc implants, and they did not require any additional therapy as the IOP remained within the normal range after FAc. Three patients underwent cataract surgery within two years after FAc implantation. It should be mentioned that a mild to advanced cataract was already diagnosed before FAc implantation in all three patients, and both IOP elevation and the development of cataracts represent common complications of chronic uveitis. One patient developed hypotension (3 mmHg) on the same day of FAc injection; the IOP spontaneously recovered after a few days. A pseudophakic patient experienced a spontaneous dislocation of the FAc implant into the anterior chamber. The implant had to be removed surgically and was placed into the vitreous chamber again.

### 3.3. Visual Acuity before and after FAc Implantation

**Figure 1:**
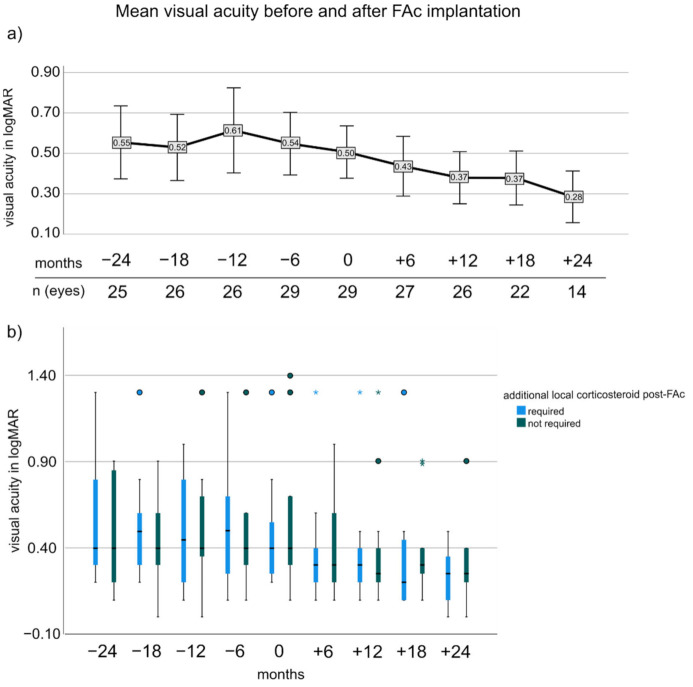
Visual acuity before and after FAc implantation. (**a**) shows the mean visual acuity and the 95% confidence interval (error bars) for the entire cohort. (**b**) visual acuity was stratified in patients with and without additional local corticosteroid injections after FAc implantation. The box represents the interquartile range(middle 50% of the data) with median (bold line); outliers are plotted as dots and extreme outliers as asterisks (defined as more than 3 × interquartile range away from the box).

For the entire cohort, VA improved from −24 months to 18 months after FAc implantation (0.55 ± 0.44 vs. 0.37 ± 0.31; *p* = 0.007, adj.*p* = 0.021) (Figure 1a). −12 months VA was not significantly different from baseline (*p* > 0.05) and VA improved in the first year after FAc implantation (0 months: 0.50 ± 0.34 vs. 12 months: 0.37 ± 0.32, *p* = 0.009, adj.*p* = 0.021). Subgroup analysis showed that mean VA at −12, 0 and 12 months was not significantly different in patients who required additional CS treatments after FAc (−12 months: 0.62 ± 0.57 (no CS) vs. 0.59 ± 0.48 (CS required), baseline: 0.45 ± 0.29 vs. 0.56 ± 0.40 and 12 months: 0.36 ± 0.29 vs. 0.38 ± 0.36, respectively (for all: adj.*p* > 0.05) (Figure 1b).

### 3.4. Central Retinal Thickness before and after FAc

**Figure 2:**
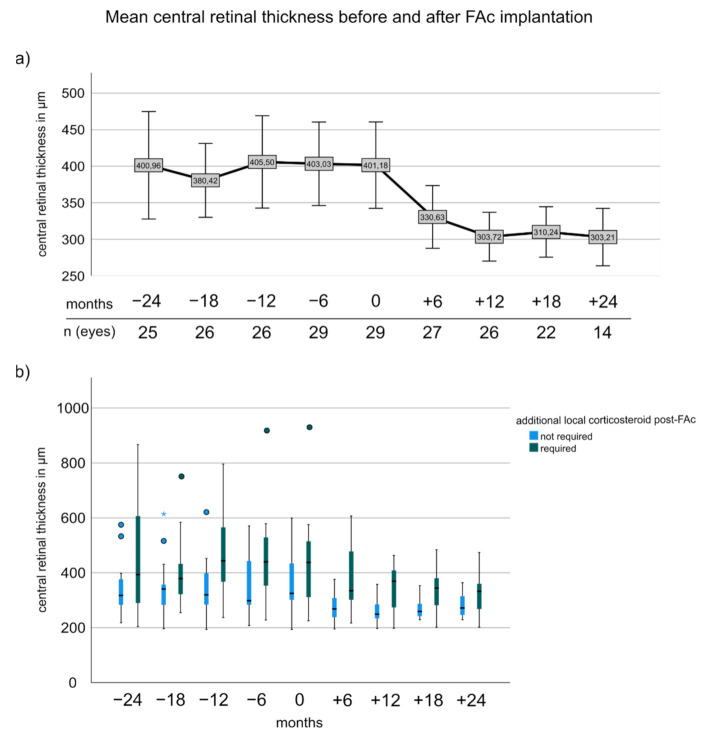
Central retinal thickness before and after FAc implantation. (**a**) Shows the mean central retinal thickness and the 95% confidence interval. (**b**) Boxplots illustrating the changes of CRT in patients with and without additional local corticosteroid injections after FAc implantation. The box represents the interquartile range (middle 50% of the data) with median (bold line); outliers are plotted as individual points.

Central retinal thickness decreased significantly after FAc implantation (Figure 2a) as shown by the pairwise comparison between −24 months and 18 months (400.96 ± 175.10 vs. 310.23 ± 76.66; *p* = 0.008; adj.*p* = 0.016). CRT at −12 months and 0 months were not significantly different (*p* > 0.05), but between 0 and 12 months (401.17 ± 151.78 vs. 303.72 ± 81.00, *p* < 0.001; adj.*p* = 0.003). Boxplots revealed that patients who required adjunctive corticosteroids after FAc implantation had higher CRT with significant differences at 6, 12, and 18 months (6 months: 275.29 ± 54.50 vs. 390.23 ± 121.51 (no CS vs. CS required); 12 months: 263.30 ± 44.82 vs. 347.50 ± 90.03 and 18 months: 269.50 ± 38.62 vs. 347.27 ± 85.10; *p*-values ranged from 0.006 to 0.027, and adj.*p*-values ranged from 0.018 to 0.032) (Figure 2b). At baseline, CRT was not different between subgroups (365.20 ± 111.03 vs. 442.69 ± 184.30, *p* = 0.235).

### 3.5. Choroidal Vascularity Index before and after FAc Implantation

**Figure 3:**
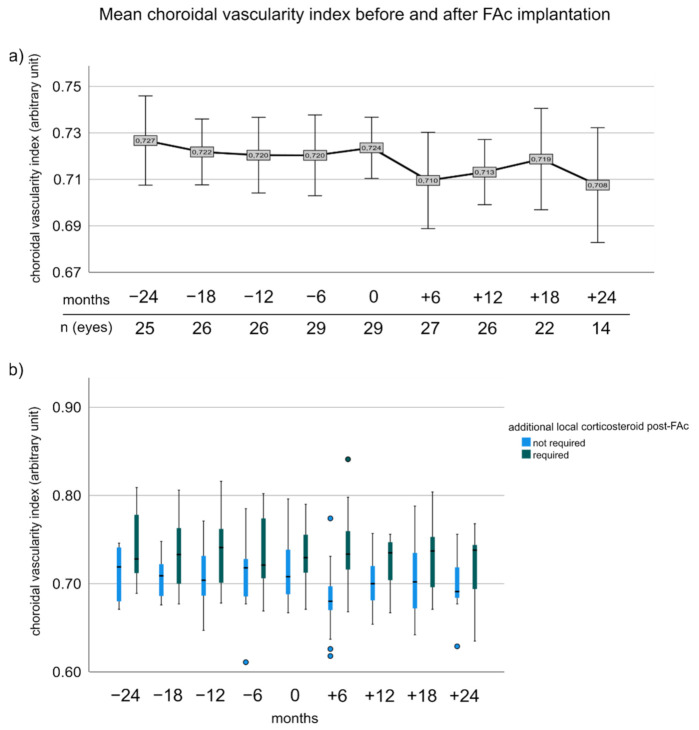
Choroidal vascularity index before and after FAc implantation. (**a**) Shows the mean CVI before and after FAc implantation for the entire cohort. Error bars represent the 95% confidence interval. (**b**) Boxplots illustrating the changes of CVI in patients with and without additional local corticosteroid injections after FAc implantation. The box represents the interquartile range (middle 50% of the data) with median (bold line); outliers are plotted as individual points.

There was a tendency of reduced CVI after FAc injection (Figure 3a). Pairwise comparison between −24 and 18 months and −12 months to baseline showed no significant difference in the entire cohort (−24 to 18 months: 0.727 ± 0.040 vs. 0.719 ± 0.046; *p* > 0.05, −12 months to baseline: 0.720 ± 0.040 vs. 0.727 ± 0.033; *p* > 0.05), but CVI significantly improved 12 months after FAc injection (0.723 ± 0.034 vs. 0.713 ± 0.033, *p* = 0.048; adj.*p* = 0.036). Patients who needed additional CS after FAc seemed to have a tendency of higher CVI across the entire observation time with significant mean differences at −18 months and 6 months (−18 months: 0.707 ± 0.022 vs. 0.736 ± 0.040, *p* = 0.034; adj.*p* = 0.248 and 6 months: 0.704 ± 0.033 vs. 0.724 ± 0.032, *p* = 0.006; adj.*p* = 0.027) (Figure 3b).

### 3.6. Ellipsoid Zone Reflectivity Ratio before and after FAc Implantation

**Figure 4:**
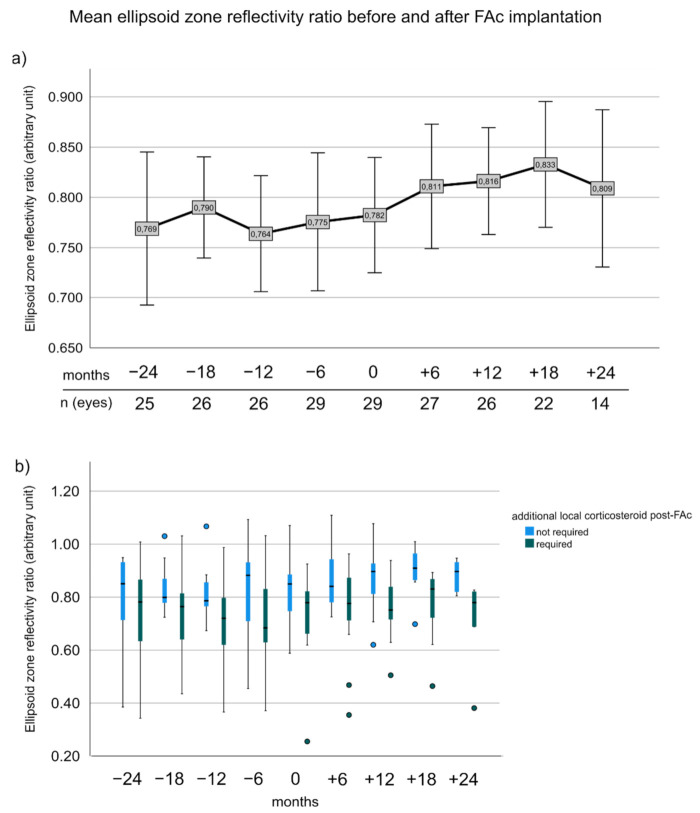
Ellipsoid zone reflectivity ratio before and after FAc implantation. (**a**) Shows the mean EZR before and after FAc implantation for the entire cohort. Error bars represent the 95% confidence interval. (**b**) Boxplots illustrating the changes of EZR in patients with and without additional local corticosteroid injections after FAc implantation. The box represents the interquartile range (middle 50% of the data) with median (bold line); outliers are plotted as individual points.

Pairwise comparison between EZR from −24 months to 18 months (0.769 ± 0.175 vs. 0.833 ± 0.133, *p* > 0.05) was not significant, but EZR from the entire cohort was overall significantly different from −12 to 12 months (0.764 ± 0.138 vs. 0.816 ± 0.130, *p* = 0.002, adj.*p* = 0.016) (Figure 4a). Patients who needed additional CS after FAc had significant worse EZR at 12 and 18 months (12 months: 0.754 ± 0.115 vs. 0.874 ± 0.118; 18 months: 0.901 ± 0.926 vs. 0.771 ± 0.138, *p*-values ranged from 0.018 to 0.022, adj.*p*: 0.054–0.058). At baseline, EZR was not significantly different between subgroups (0.733 ± 0.171 vs. 0.821 ± 0.122, *p* = 0.119) (Figure 4b).

### 3.7. Change of CVI and Number of CS Injections Prior to FAc Correlated to the Need of Additional CS after FAc

The number of CS per year after FAc implantation was significantly correlated to the number of CS per year prior FAc (Pearson’s correlation coefficient (R): 0.49, *p* = 0.006), CVI change from baseline to month 6 after FAc (R: −0.41, *p* = 0.040) and CRT at baseline (R: 0.38, *p* = 0.044). EZR at baseline was at the border of significance (R: −0.36, *p* = 0.056), while VA and CVI at baseline, as well as changes of VA, EZR, and CRT from baseline to 6 months, were not significantly linearly correlated to the number of CS required after FAc (*p* > 0.05). Parameters with a significant correlation represented putative predictors for the number of CS required after FAc. Therefore, they were tested in a generalized Poisson regression model for their predictive values (Table 2). CS per year before FAc and CVI change from baseline to 6 months were significant predictors for the number of CS needed after FAc (*p* = 0.017 and *p* = 0.004). The model indicated that an increase in CS per year before FAc was predictive for an increase in CS required after FAc (OR = 2.65). In contrast, a decrease in CVI from baseline to 6 months was inversely correlated (OR = 0.82), suggesting that the more the short-term CVI reducing effect of FAc, the less is the chance to get additional CS after FAc.

## 4. Discussion

In this study, the morphological and functional parameters before and after FAc implantation for up to 2 years were assessed, and putative predictors that were associated with the number of CS injections required after FAc implantation were explored. The more CS was needed before FAc, the higher the chance for additional CS after FAc implantation. Furthermore, our results suggest that a steeper decrease in CVI reduces the likelihood of additional CS after FAc. In agreement with previous reports about the efficacy of FAc in non-infectious uveitic macular edema, a decrease in CRT and improvement of VA were observed for up to 2 years after FAc implantation in our cohort [28,34,35]. Our study confirmed that both DEX and FAc implants are similarly effective in improving VA [36]. There was no difference in VA between patients who required additional CS after FAc and those who were CS-free. Notably, OCT parameters such as CRT, CVI, and EZR improved significantly in those patients who did not need additional CS after FAc, thus underlining the importance of implementing additional morphological and functional parameters to support decision making.

The literature on OCT biomarkers in UME is limited. To the best of our knowledge, this is one of the first studies describing long-term changes of OCT biomarkers, and putative predictors for treatment response in UME treated with FAc. Ciulla et al. recently published a post-hoc analysis of two phase 3 clinical trials on UME treated with injectable triamcinolone acetonide, in which the predictive value of EZ integrity and presence of fluid at baseline on therapeutic response was investigated. The authors concluded that anatomical responses preceded VA improvement, and both pre-treatment EZ integrity and subretinal fluid were predictive for improved treatment outcomes [37]. In another report, Cicinelli et al. proposed that the morphologic response to previous dexamethasone implants might anticipate the response to subsequent FAc in diabetic macular edema and the need for additional therapy [38,39]. The same question arises for UME as patients with both disease entities usually receive multiple dexamethasone implants prior to FAc implantation [34,36]. Our subgroup analysis was divided into two groups: patients who did not require any additional corticosteroids after FAc and those that did, as the need for additional CS treatment remains difficult to predict but plays a major role in patients’ decision for FAc implantation and in disease management in general.

An increase in CVI at the time of active uveitis disease and subsequent reduction after resolution of inflammation have been reported by several authors for intermediate and posterior uveitis [40,41]. Overall, lower CVI was observed in quiescent or resolved uveitis. Arrigo et al. conducted a prospective 1-year study to evaluate the inflammatory profiles of DME patients that included quantification of hyperreflective foci and CVI. The authors concluded that the inflammatory profile was useful in distinguishing between good and poor responders. Good responders were characterized by more hyperreflective foci and lower CVI [42]. Similarly, in our cohort, an overall lower CVI and greater decrease in CVI after FAc was observed in patients who did not require additional CS injection. These results suggested that short-term CVI response to FAc provides context to individual inflammatory profile to some extent and might be a useful parameter for monitoring therapy. While CVI has been proposed as an OCT biomarker of acute inflammation, ellipsoid zone integrity was regarded as a parameter that can be associated with longer recovery time. Studies about EZ recovery after retinal surgery or in uveitis reported about EZ recovery that was ongoing even after 12 months [43,44]. This could account for the late onset of improved EZR after 12 months. However, baseline EZR and EZR change 6 months after FAc were not significantly different between subgroups. Therefore, as a baseline predictor for treatment response, EZ might not be as suitable as CVI.

While EZR and CRT were not different in subgroups at baseline, EZR and CRT were substantially better in patients who did not require adjunctive CS in the follow-up time. We speculate that the continuous low-dose drug release kinetics of FAc provided it controls the local inflammatory environment without additional CS leads to sustained inflammatory control and reduced fluctuations in macular edema, thus presumably having a positive effect on photoreceptor function over time [45]. However, despite better CRT and EZR after FAc implantation in patients that did not require adjunctive CS, VA outcomes were comparable to those treated with implant and did not require additional CS treatment. As uveitis is a chronic disease with recurrent inflammation, the potentially positive long-term effect of improved EZR and CRT under FAc needs further investigation.

Limitations of this study include the small sample size, varying observation periods, and its retrospective design. Furthermore, VA and CRT inclusion criteria were not stringent, thus leading to a higher variation of functional and morphological parameters within the cohort. On the other hand, the aim of this study was to provide an analysis that reflects outcomes achieved in real-world practice. Due to the higher variation of OCT and VA parameters prior to FAc implantation and some missing values at various time points, regression analysis only included parameters at baseline and quantitative changes from baseline to 6 months.

It would be desirable to stratify the cohort according to the different CS received prior to FAc implantation (subconjunctival or periocular injection of triamcinolone acetonide and intravitreal DEX implants) as triamcinolone acetonide applied in different ways and intravitreal DEX have distinct pharmacokinetic profiles [46]. However, the small cohort size, the considerable inter-individual variation of injection frequency, and the different drugs administered meant it was too challenging for a more refined stratification. Hence, all three types of local CS counted equally into the quantification of received CS.

The strengths of this study include: the evaluation of multiple OCT biomarkers, including ellipsoid zone reflectivity ratio and choroidal vascularity index that have not been described in non-infectious UME FAc-treated eyes, the inclusion of one eye of each patient, and the long duration of follow-up.

## 5. Conclusions

In conclusion, our study sheds light on long-term functional and anatomical outcomes in UME treated with the FAc implant. The number of CS required prior to FAc injection predicted the need for additional CS after therapy with the implant. In contrast, a higher decrease in CVI at 6 months after FAc therapy commenced was negatively correlated to the number of additional CS needed after the implant was given. These parameters may anticipate the need for adjunctive CS. Further studies are needed to further clarify the role of OCT biomarkers such as CVI in the management of UME.

## Figures and Tables

**Table 1:** Patients’ characteristics.

Demographic Characteristics	*n* (in %) or Mean (±Standard Deviation)
Numbers of patients (one eye per patient)	29
Right eye as study eye	11 (38%)
Age at FAc implantation (in years)	60.03 ± 16.01
Mean observation time (in years)	3.51 ± 0.51
Male	10 (34%)
Disease duration until FAc implantation (in years)	8.55 ± 6.20
Uveitis classification	Numbers of eyes (in %)
Uveitis anterior with ME	2 (7%)
Uveitis intermedia with ME	18 (62%)
Uveitis posterior with ME	5 (17%)
Panuveitis with ME	4 (14%)
Etiology of non-infectious uveitis	Numbers of eyes (in %)
Idiopathic	18 (63%)
Sarcoidosis	3 (11%)
Birdshot retinopathy	2 (7%)
Ocular tuberculosis (non-infectious when treated)	1 (3%)
Multifocale chorioretinitis and panuveitis	1 (3%)
Vogt-Koyanagi-Harada syndrome	1 (3%)
Acute zonal outer occult retinopathy (AZOOR)	1 (3%)
Multiple sclerosis	2 (7%)
Lens and vitreous status, n (%)	Numbers of eyes (in %)
Pseudophakic	26 (89%)
Vitrectomized eyes	4 (14%)
Local treatments (dexamethasone implants or peribulbar corticosteroids)	Mean (±standard deviation)[range]
Total number of local corticosteroid injections within 2 years prior to FAc	4.52 ± 1.90 [1; 9]
Total number of local corticosteroid injections in the 2 years after FAc	1.45 ± 1.94 [0; 6]
Local corticosteroid injection per year prior to FAc	2.40 ± 0.88 [1.0; 4.5]
Local corticosteroid injection per year after FAc	0.94 ± 1.33 [0; 5]

ME = macular edema, FAc = fluocinolone acetonide.

**Table 2:** Overview of covariates in the generalized Poisson regression model.

Covariates	Coefficient	Standard Error	OR	95% CI	*p*-Value
CS per year before FAc	0.97	0.37	2.65	1.21; 5.78	0.017 *
EZR at baseline	−0.49	0.32	0.61	0.31; 1.21	0.148
CRT at baseline	−0.40	0.29	0.67	0.36; 1.23	0.183
CVI reduction from baseline to 6 months	−0.20	0.05	0.82	0.73; 0.93	0.004 *

OR: odds ratio, CI: confidence interval, * *p* < 0.05.

## Data Availability

All available data generated or analyzed during this study are included in this published article.
